# Ovarian reserve may be compromised after adnexal surgery: Are we sufficiently fertility- focused in our surgical training?

**Published:** 2016-06-27

**Authors:** GS Kalra, S Campbell, G Nargund

**Affiliations:** CREATE Fertility, 150 Cheapside, St Paul’s, London EC2 V 6ET.

**Keywords:** Ovarian reserve, adnexal surgery, salpingectomy, ovarian cystectomy, fertility focus, surgical training

## Abstract

There is a general trend towards delay in childbearing age amongst women. The ovarian reserve clearly falls with increasing age and the impact is greater with advancing age, particularly from late 30s. Presence of other risk factors can increase the risk of subfertility. A large number of women are exposed to pelvic surgery for various reasons, both elective and emergency. There is evidence that some of the pelvic surgery performed around ovaries and tubes has a negative impact on the ovarian reserve and in turn may cause a decline in woman’s ability to conceive. A fertility-sparing focus on all pelvic surgery is likely to prevent further decline in ovarian reserve for women who are already at higher risk. Such focus seems to be currently lacking. It is proposed that integrating fertility-sparing focus to structured gynaecological surgical training will benefit women.

## Delay in childbearing

A definite trend of delay in childbearing age in females has been witnessed over the last few decades, largely in the developed world. The trend seems to be extending to less developed countries and seems to have been concurrent with increasing education amongst women and their greater participation in the work force (Oppenheimer and Blossfeld, 1995). In the Netherlands the mean age of women at first childbirth has gone up from 24.8 in 1970 to 28.6 in 1990, while in the UK the mean age of women at first childbirth increased from 27.3 to 29.1 from 1990 to 2000 (European Council Recent demographics, 2003).

## The reproductive ageing in human female – An ovarian concept

It is a widely prevalent concept that reproductive ageing in human females is primarily a function of ovarian ageing. This in turn is understood to mean a fall in the quantity and quality of oocytes with increasing age. It is known that the human female is endowed with a finite number of oocytes, which peaks at around 18-22 weeks of gestation. This oocyte pool decreases to about one million at birth and to about 300,000 at puberty (Gougeon, 1986; Faddy and Gosden, 1992, 1996). There is an accelerated loss from 37-38 years onwards (Faddy and Gosden, 1992). The total number of follicles remaining in the ovaries, amongst other factors decides the age of menopause in women when only a few hundred up to a thousand oocytes are left (Fauser and Van Heusden, 1997).

**Fig. 1:**
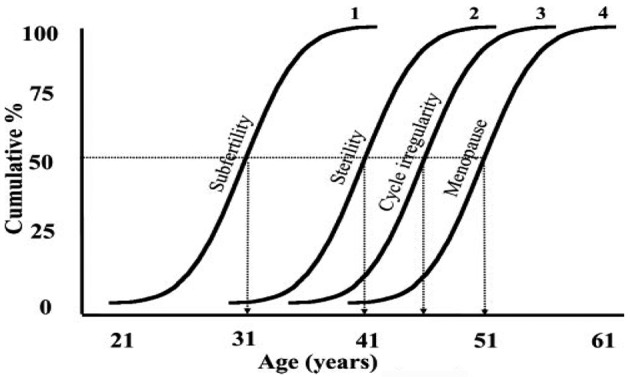
— Variations in mean age of women at the occurrence of specific stages of ovarian ageing. (Broekmans FJ et al., Hum Reprod Update 2006, Background data from te Velde and Pearson, 2002).

The age of optimal fertility in a human female is considered to be 18-30 years, after which there is a decline in the ovarian function leading to the final event of menopause through progressive stages illustrated in Figure 1. Te Velde and Pearson plotted these stages with Gaussian distribution of age in a cumulative fashion and show that the mean age of the start of reduced fertility is 31 years, the start of low fertility is 41 years, and the start of cycle irregularity is 45-46 years and mean age of menopause is 51 years (te Velde and Pearson, 2002).

Endometrial ageing seems not to follow chronological ageing, as the success rate of IVF in older women with donor eggs does not decline (Legro et al., 1995; Check et al., 2000). Experiments involving unilateral oophorectomy in mice have shown that by causing about 50% reduction in the number of ovarian follicles these mice suffered from earlier subfertility, cycle irregularity and menopause. The rate of occurrence of oocyte aneuploidy was also higher. Although this has not been sufficiently studied in humans, similar findings are observed in human females (te Velde and Pearson, 2002). The stages of reproductive ageing are therefore a function of total number of follicles present at any time.

## The fall in oocyte quality follows decreasing oocyte quantity

The occurrence of higher rate of oocyte aneuploidy and increasing rates of miscarriages with age has been extensively studied. There is a significant increase in incidence of trisomy 21 with age, which is much higher after 40 years of age. Chromosome studies done on spontaneous abortions have shown that about 80% of all trisomy 21 fetuses end in spontaneous abortion (te Velde and Pearson, 2002). Other trisomies not seen in live born have also been observed in chromosome studies of spontaneous abortions and show an age related increase. These findings suggest that the best oocytes are probably recruited in a woman’s earlier years leaving poorer quality oocytes to grow with advancing age (Nikolaou and Templeton, 2003). There seems therefore to be a relationship between the number of oocytes left in the ovary and the quality of oocytes at any age. Thus the ovarian ageing or the age dependent loss of female fertility is considered to be a combination of decline in quantity and the quality of oocytes (Kalra, 2009).

## Relation between increasing age and decreased success rate of IVF

The success rate of IVF treatment in the form of live birth rate peaks between 25-30 years of age with small decline below and a sharp age related decline in older women (Templeton, 1996; Broekmans et al., 2006). The age related decrease in success rate of IVF is more significant after 35 years age. There is also an age related decline in implantation rate per embryo (Broekmans et al., 2006).

## Ovarian reserve after benign pelvic gynaecological surgery

The bulk of benign pelvic gynaecological surgery on the adnexae constitutes mainly ovarian cystectomy for benign tumours (including cystic teratomas and endometriomas) and salpingectomy for ectopic pregnancy. There is scarcity of randomized trial evidence to clarify the effect of surgical insult to the vasculature and removal of ovarian tissue on ovarian reserve, ovarian response to controlled ovarian hyperstimulation and success rate of subsequent ART. A study of women who had a previous removal of benign ovarian cysts (including endometriomas and dermoid cysts) reported that a significantly lower number of follicles and oocytes were retrieved from the operated ovary compared to the contralateral ovary during IVF treatment (Nargund, 1996). The number of follicles and oocytes retrieved were lowest after endometriotic cyst excision. In 2003 Somigliana confirmed Nargund’s results by studying the ovarian response during IVF in women who had had an excision of endometrioma in the past (Somigliana et al., 2003). By comparing the ovarian response to controlled ovarian hyperstimulation between the operated and non-operated ovary they found a significant difference in the number of follicles and oocytes retrieved in the operated ovary irrespective of size of endometriomas and (Somigliana et al., 2003). Ho et al. in 2002 reported similar results. However Donnez et al. in 2001 and Loh et al. in 1999 reported no difference (Donnez et al.; Loh, 1999). It is to be noted that Donnez et al. used cyst wall vapourisation with plasma jet rather than excision. Indeed, it is difficult to conclude whether this is the effect of surgery only as it has been shown that the ovarian stroma around endometriotic cysts seems to be showing microscopic stromal implants and reduced follicular number and activity, which is not seen in benign cysts like mature teratomas and cystadenomas (Maneschi et al., 1993; Muzii, 2002). However it is important to note that endometriotic cyst surgical removal seems to lead to better outcome compared to ablation, in relation to symptom control, recurrence rate as well as rate of spontaneous pregnancy post treatment (Hart, Cochrane 2005). The blood supply of the ovary is very closely related to the Fallopian tube. Any surgery around the tube carries a potential risk of damaging the ovarian blood supply leading to risk of impairment of the ipsilateral ovarian function. This has been confirmed in some animal studies (Byeth et al., 1981, McComb and Delbeke, 1984) but human studies are scarce.

**Fig. 2:**
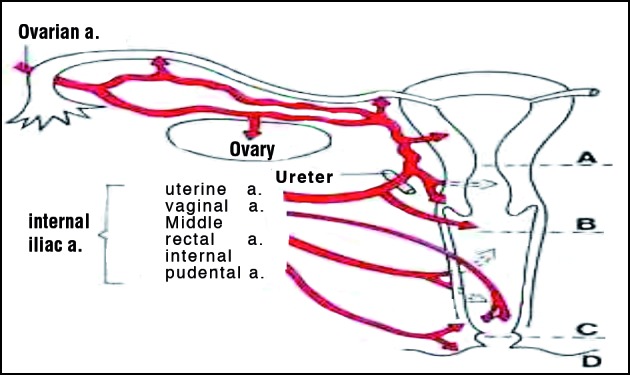
— Ovarian vascular supply and the potential risk of disruption during salpingectomy (figure taken from google images).

## Adnexal surgery for ectopic pregnancy

Surgery for tubal ectopic pregnancy constitutes a large proportion of pelvic surgery and is increasingly performed by the laparoscopic technique (Taheri, 2014). Laparoscopic approach is convenient for the patient and leads to reduced costs as well as reduced risk of complications compared to open surgery (Jönsson and Zethraeus, 2000; Hajenius, 2009).

Up to 95% of ectopic pregnancies are tubal and most of these are in the distal half of the tube (Clausen, 1996). Salpingectomy is the recommended procedure when the contralateral tube appears to be normal (Clausen, 1996; Yao and Tulandi, 1997). A recent open-label, multicenter, international, randomised control trial comparing salpingotomy with salpingectomy for ectopic pregnancy in presence of healthy contralateral tube and using spontaneous conception after 36 months as primary outcome, provided robust evidence to support salpingectomy (Mol et al., 2014). There is similar supporting evidence in favour of salpingectomy when outcomes are related to assisted reproduction (Odesj et al., 2015).

Lass et al. (1998) showed that after salpingectomy for ectopic pregnancy fewer follicles developed as well as fewer oocytes retrieved from ipsilateral side in IVF cycles. Xi et al. in 2012 showed that women undergoing controlled ovarian hyperstimulation after laparoscopic salpingectomy needed significantly higher total dose of gonadotropinsn (Xi et al., 2012). Grynnerup et al. (2013) showed lower AMH levels in women post salpingectomy prior to starting IVF treatment.

Prophylactic salpingectomy performed for hydrosalpinx however has been shown to be of benefit and seems to improve the outcome of IVF (Strandell, 2001). The suggested hypothesis for this is that the presence of swollen tubes with toxic stagnating fluid content is more damaging than the risk of tubal surgery (Strandell, 2002). Therefore overall it is beneficial to remove the hydrosalpinx prior to IVF.

The ovarian blood supply is closely related to the fallopian tube and there is potential risk of reducing ovarian vascularity while performing surgery in the vicinity. Commonly used methods of performing laparoscopic salpingectomy are the following with first two being the commonest-

- Endoscopic pre-tied loop. This brings the fallopian tube including the ectopic pregnancy through a pre-tied endoscopic surgical loop, followed by tightening of the loop into a knot around the fallopian tube and surgical resection is performed. This method can be uncritical and likely to include some of the surrounding mesosalpinx into the loop which may lead to damage to the adjacent ovarian blood supply (Hajenius, 2009; Capmas, 2014).
- Electrosurgery (monopolar or bipolar diathermy) is used to coagulate the vessels in mesosalpinx and resect the fallopian tube with scissors (Capmas, 2014). Uncritical use of diathermy, resection of mesosalpinx and lack of fertility conserving focus can be deleterious to the ovarian vasculature.
- Ultrasonic scalpel uses ultrasonic vibrations (up to 55 KHz) instead of electrosurgery to cauterize and cut at the same time with one instrument. Technique otherwise is largely the same as with electrosurgery with similar risks to the ovarian vasculature, if fertility focus is lacking.
- Endoscopic automatic stapling devices can be used from the proximal end to the distal end to staple and cut the tube from mesosalpinx. The lack of fertility focus as above is likely to be damaging to ovarian vasculature.

One can therefore assume that during adnexal surgery, there is a risk of impact on ovarian blood supply and the effect may be more marked in women with other risk factors for reduced ovarian reserve e.g. age > 38 years, previous poor ovarian response, heavy smoking, previous unilateral oophorectomy, chemotherapy, radiotherapy and severe endometriosis.

With a large amount of benign pelvic gynaecological surgery being performed on women of fertile age, it is very important to be aware of any intervention that can potentially impair the fertility.

## Fertility focus to pelvic surgery needs to be integral to training.

The technique and equipment used for doing the salpingectomy depends on the surgeon and the unit. There is a lack of standardization of the technique used for laparoscopic salpingectomy. The United Kingdom national survey of trends in ectopic pregnancy management in 2014 suggested that 57% of all ectopic pregnancies are operated laparoscopically (Taheri, 2014). Laparoscopic salpingectomy is likely to be the first laparoscopic procedure a trainee is exposed to. All specialists trained in gynaecology and working on call are expected to be able to perform a laparoscopic salpingectomy safely (Larsen et al., 2008). There are no specific OSATs (Objective Structured Assesment for technical skills) for laparoscopic salpingectomy as a part of Obstetrics and Gynaecology training curriculum in the United Kingdom. It is authors’ experience that generally no specific fertility conserving consideration is given to the procedure, leading to potential risk of short or long-term impairment of ovarian vasculature and ovarian reserve.

Women who have other risk factors for low ovarian reserve and response will be more prone to suffering further decline to their fertility health following salpingectomy for ectopic pregnancy or removal of ovarian cysts. We believe there is a need to routinely assess ovarian reserve of women in fertile age who wish to have children and are admitted with adnexal masses and ectopic pregnancy. We highly recommend that a fertility conserving focus to pelvic surgery is added to the curriculum already at medical school level and later at speciality training level.
